# Residual Fatigue and Cognitive Deficits in Patients After Leucine-Rich Glioma-Inactivated 1 Antibody Encephalitis

**DOI:** 10.1001/jamaneurol.2021.0477

**Published:** 2021-03-29

**Authors:** Sophie N. M. Binks, Michele Veldsman, Ava Easton, M. Isabel Leite, David Okai, Masud Husain, Sarosh R. Irani

**Affiliations:** 1 Oxford Autoimmune Neurology Group, Nuffield Department of Clinical Neurosciences, Oxford, England; 2 Department of Neurology, Oxford University Hospitals NHS Foundation Trust, Oxford, England; 3 Department of Experimental Psychology, University of Oxford, Oxford, England; 4 The Encephalitis Society, Malton, North Yorkshire, England; 5 Honorary Fellow, Depatment of Clinical Infection, Microbiology and Immunology, University of Liverpool, Liverpool, England; 6 Department of Neuropsychiatry, Maudsley Outpatients, Denmark Hill, Maudsley Hospital, London, England; 7 Nuffield Department of Clinical Neurosciences, Oxford, England

## Abstract

This study evaluates residual fatigue and cognitive defects in patients after leucine-rich glioma-inactivated 1 antibody encephalitis.

Leucine-rich glioma-inactivated 1 antibody encephalitis (LGI1-Ab-E) typically affects older men who present with prominent amnesia and frequent seizures^1,2,3^ and often shows a marked short-term improvement with immunotherapies.^4^ In particular, seizure cessation occurs within just a few weeks. However, only traditional cognitive domains have been investigated as longer-term outcomes, with improvements in cognition described as “not good enough.”^5^ Here, motivated by patient feedback and our clinical observations, we aimed to quantify the residual deficits observed after LGI1-Ab-E across several functional domains.

## Methods

Participants were recruited to this cross-sectional study from previous cohorts,^4^ author clinics, or via the Encephalitis Society and assessed by neurologist interview and a battery of tests measuring:

1. Cognition: Addenbrooke’s Cognitive Examination (ACE), Mini-Mental State Examination (MMSE), and Frontal Assessment Battery (FAB)
2. Affective symptoms: Hospital Anxiety and Depression Scale (HADS)
3. Clinician-rated disability: modified Rankin Scale (mRS) and the Clinical Assessment Scale in Autoimmune Encephalitis (CASE)
4. Fatigue: Fatigue Scale for Motor and Cognitive Function (FSMC) and Modified Fatigue Impact Scale (MFIS).

All patients gave written informed consent (Research Ethics Committee approval 16/YH/0013).

## Results

Clinical data were gathered from 60 patients with LGI1-Ab-E, assessed at a median of 41 months (range, 4-179 months) after symptom onset (Table). From peak illness to post illness, a marked fall in disability was noted using both the CASE (median [SD] score of 6 [3.4] to 2 [1.7]) and mRS (median [SD] score of 3 [1.1] to 2 [1.1]; Figure, A; both *P* < .001), with 81% (n = 48 of 59) showing a “good” functional outcome (mRS ≤2). However, only 4 of 27 (15%) of those in employment at diagnosis returned to their premorbid role (Table). The median age of those medically retired or transitioning to a less demanding role was 56 years (range, 46-70 years), representing a reduction of 10 years’ fully productive working life in the United Kingdom. Consistent with this vocational effect, more detailed clinical testing captured widespread deficits.

**Table.** Cohort Demographics and Clinical Features of 60 Patients With Leucine-Rich Glioma-Inactivated 1-Antibodies

Demographics	Median (range)^a^	Patients, No./total No. (%)^b^
Age at onset, y	64 (44-86)	60 (100)
Age at assessment, y	70 (44-92)	60 (100)
Assessment post onset, mo (range)	41 (4-179)	60 (100)
Female	NA	20/60 (33)
Clinical syndrome at presentation		
Epilepsy	NA	10/60 (17)
Encephalitis	NA	48/60 (80)
Morvan syndrome	NA	1/60 (2)
Other (stroke)	NA	1/60 (2)
Clinical features at presentation		
Any seizure	NA	59/59 (100)
Faciobrachial dystonic seizures	NA	42/59 (71)
Focal onset seizures	NA	39/59 (66)
Generalized seizure	NA	17/59 (29)
Amnesia	NA	49/59 (83)
Tumor	NA	10/59 (17)
Therapeutic and medical history		
Ever had immunotherapy	NA	53/59 (90)
Steroids	NA	52/59 (88)
Intravenous immunoglobulins	NA	25/54 (46)
Plasma exchange	NA	18/55 (33)
No. of weeks to IT	16 (3-250+)	57 (NA)
Functional status		
Current mRS, mean (range)	1.6 (0-4)	59 (NA)
mRS>2	NA	11/59 (19)
Employment status when assessed		
Employed, same role	NA	4/58 (7)
Employed, reduced role	NA	12/58 (21)
Medically retired because of LGI1-Ab-E	NA	11/58 (19)
Retired at onset or other cause	NA	31/58 (52)

Abbreviations: IT, immunotherapy; LGI1, leucine-rich glioma-inactivated 1 antibody encephalits; mRS, modified Rankin Scale.

^a^ Empty cells reflect continuous variables.

^b^ Denominators indicate the number of patients with available data.

**Figure.**
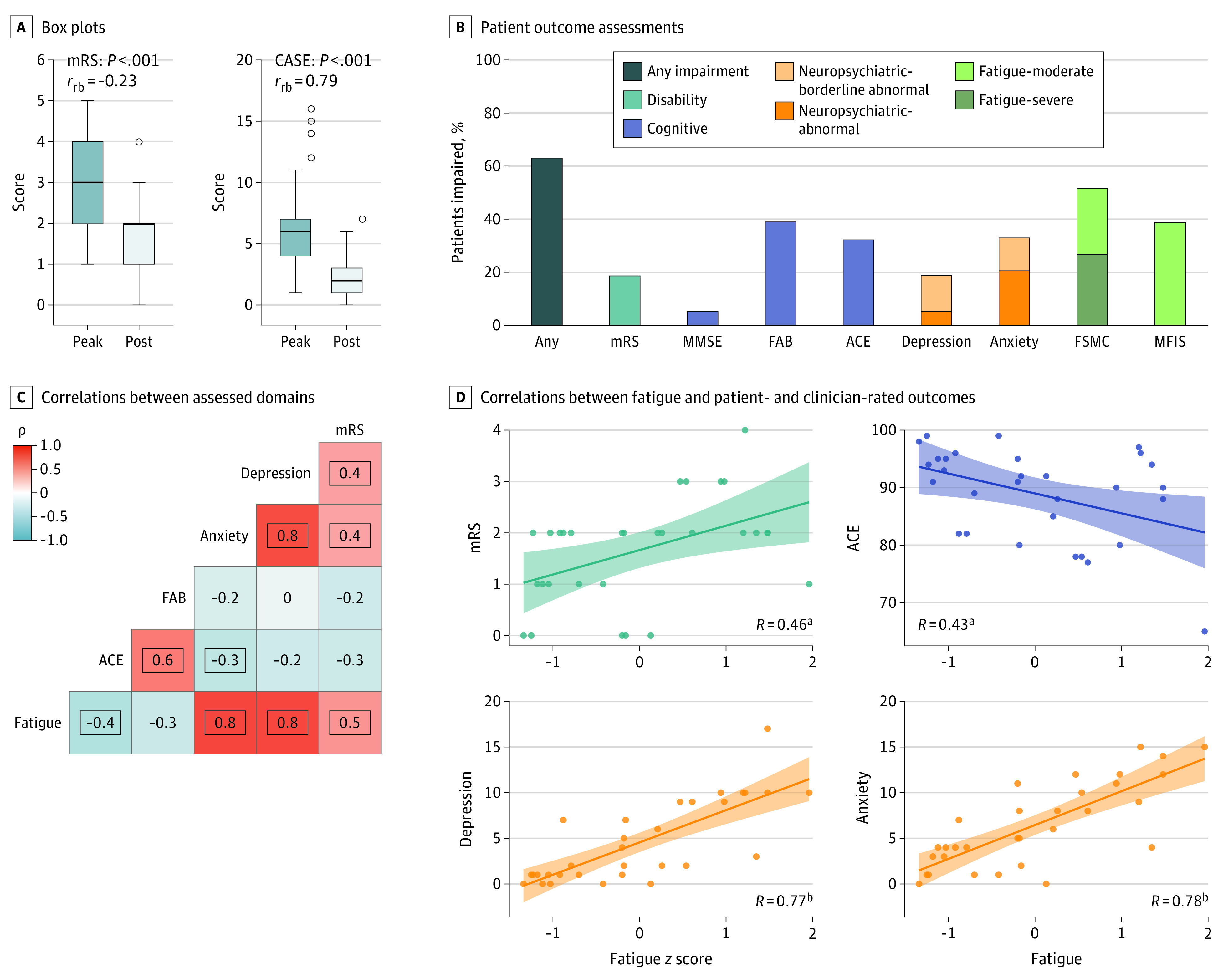
Outcomes in Patients With Leucine-Rich Glioma-Inactivated 1 Antibody Encephalitis A. Peak illness to postillness scores in physician-rated modified Rankin Scale (mRS) and Clinical Assessment Scale in Autoimmune Encephalitis (CASE). B. Results of patient outcome assessments across multiple domains. Bar graph depicting proportion of patients with abnormal scores. Shading denotes neuropsychiatric scores from Hospital Anxiety and Depression Scale (borderline abnormal or abnormal) (CASE not depicted because no normative value for healthy controls exists). Fatigue scales were introduced during the study and completed by 31 patients: those without or with fatigue questionnaires were closely matched other than a shorter duration from illness onset in the latter group (37.7 vs 75.4 months; t(51.74) = 3.270; *P* = .002). C. Single-correlation *R* values and Pearson correlation shown across outcome measures (Bonferroni-adjusted for multiple comparisons, with outlined boxes for *P* <.01). D. Graphs show correlations between fatigue *z *score (x-axes) and mRS, Addenbrooke’s Cognitive Examination (ACE), and depression/anxiety (both derived from Hospital Anxiety and Depression Scale). FAB indicates Frontal Assessment Battery; FSMC, Fatigue Scale for Motor and Cognitive Function; MFIS, Modified Fatigue Impact Scale; MMSE, Mini-Mental State Examination. ^a^*P* < .01. ^b^*P* < .001.

By comparison with age-appropriate cutoffs derived from manuals or publications, 63% of the LGI1-Ab-E cohort (n = 38 of 60) were impaired on at least 1 of cognition, mood, and fatigue (Figure, B). Cognitive testing revealed total ACE was impaired in 32% (n = 18 of 56; score <88 of 100); 16% (n = 9 of 56) attained scores less than that of healthy elderly individuals in memory, fluency, and visuospatial capabilities, whereas attention (9% impaired [n = 5 of 56]) and language abilities (5% impaired [n = 3 of 56]) were relatively spared. Of affective features, both depression (HADS-D >7) and anxiety (HADS-A >7) were present in 19% (n = 11 of 58) and 33% (n = 19 of 58), respectively. However, overall fatigue was the most common long-term deficit, detected in 52% with the FSMC (n = 16 of 31), rated as severe in 56% of these patients (n = 9 of 16) (Figure, B).

The interrelationships between these deficits revealed the strongest correlations between fatigue and both anxiety and depression (ρ = 0.78 and ρ = 0.77, respectively; *P* < .001, after Holm-Bonferroni multiple comparison corrections; Figure, C and D). In addition, extent of fatigue correlated with both the greater disability (from mRS) and poorer cognition by ACE (Figure, D).

## Discussion

Although mRS represents the most widely used outcome measure in studies of autoimmune encephalopathies, the data here indicate that despite a “good” mRS, several long-term residual deficits remain: across domains of cognition, mood, and fatigue, with a significant effect on employment status. Our cohort’s mean mRS was comparable with other LGI1-Ab-E studies,^2,3,4^ suggesting this traditional outcome measure captures only limited long-term morbidity in multiple studies. Fatigue was the most commonly impaired domain in our cohort, a novel finding in LGI1-Ab-E. This observation is closely reflected by the many patients in our clinic who volunteer fatigue as a major residual symptom. Also, it parallels findings in pediatric *N*-methyl-d-aspartate receptor antibody encephalitis, where fatigue is associated with quality of life.^6^

Overall, we continue to advocate early immunotherapy to achieve optimal clinical outcomes in patients with LGI1-Ab-E. Future studies can now also ask whether this approach mitigates the appearance of fatigue, in addition to amelioration of the other expanded long-term cognitive deficits highlighted within our study.
